# Revealing Secrets: Talismans, Healthcare and the Market of the Occult in Early Twentieth-century China

**DOI:** 10.1093/shm/hkab035

**Published:** 2021-06-08

**Authors:** Luis Fernando Bernardi Junqueira

**Affiliations:** PhD Candidate, Department of History, University College London & Wellcome Trust, UK

**Keywords:** modern China, Chinese medicine, spiritualism, occult; magic

## Abstract

This article analyses the place and value of occult arts in the healthcare market of Republican China (1912–1949). Medical historiography has long neglected the resilience of such occult arts as talismans, astrology and divination in the context of China’s search for modernity. Focusing on the production, trade, and consumption of goods and services related to talismanic healing, I give voice to Chinese occultists by investigating the formation of a ‘market of the occult’ in the Republican era. I adopt a global perspective to clarify the changes that occult healing underwent following the popularisation of new printing technologies, mass media and transnational spiritualism in early twentieth-century China. Erstwhile embraced in secrecy, the occult was now being made public. Cheap manuals, wide-circulation newspapers and book catalogues reveal that in contrast to past studies that herald the disenchantment of the world as the hallmark of Chinese modernity, occult healing did not simply survive but thrived in the face of modern science and technology.

##  

This article is about occult arts and their place in the healthcare market of Republican China (1912–1949). In 1894, the *Shanghai News*, a major Chinese newspaper, reported a case of miraculous healing which happened in the house of Mr Chen 陳, a wealthy 60-year-old man from Suzhou. Mr Chen’s son, Jinbao 金寶, at the time in his twenties, had long been afflicted by a nervous disease that herbals and acupuncture were unable to cure. One day, after hearing that a talismanic healer from Hunan was visiting Suzhou, Mr Chen rushed to invite him to see his sick son. Asking the family to place an incense altar outside the room where Jinbao was resting, the healer then wrote the patient’s name and birthday on a piece of yellow paper, burned incense, lit a candle and created three talismans. While Mr Chen kneeled in front of the altar, the healer burned the first talisman and uttered ‘Jinbao, wake up!’, after which the lad began to move in the bed. When he burned the second talisman and repeated ‘Jinbao, wake up, wake up!’, Mr Chen could hear his son getting out the bed. Finally, when the healer burned the third talisman and uttered ‘Jinbao, come here!’, the lad jumped out of the room and enthusiastically responded ‘Here I am!’. Jinbao, the newspaper reports, was healed as if his disease had never existed.^1^

Almost half a century later, just before the beginning of the Communist rule, accounts of miraculous healing by talismans continued to resonate through the Chinese press. In an article titled ‘Healing Disease? Cheating People? Talismanic Healing and the Like’, Zhu Min 朱旻, a columnist for the magazine *Weekly Phenomena*, published his views and personal experience with talismanic rituals. The motivation for writing this piece, Zhu argues, was triggered after he saw a number of newspaper advertisements posted by people looking for an ‘authentic talismanic healer’. The piece informs us that the first time Zhu saw a talismanic healer in action was on a street close to his middle school in the late 1930s. Surrounded by a curious crowd, the healer unfolded a rag onto the floor, upon which he placed tools for writing talismans. A man then came forward, complaining of a stomach-ache that no physician had been capable of treating thus far. After asking the man to take off his shirt and point to where he felt pain, the healer took out a paper figurine and pierced it with a long needle in the exact same spot that corresponded to the man’s abdomen. After burning dried mugwort onto it, the healer then asked the man if he was still feeling pain, to which the man shook his head in bewilderment—to everyone’s astonishment. What struck the crowd the most, however, was when the man lifted his shirt: a reddish dot appeared in his abdomen at the same place where the healer had applied needles and mugwort to the paper figurine.^2^

Although such accounts ‘would make some people laugh’, the article states, ‘there are many things that science cannot yet explain’, and we should be cautious not to jump to conclusions too quickly. Referring to an article on hypnosis published in the *New York Times* a few months earlier, Zhu then champions the idea that Chinese techniques of talismanic healing work in the same way as Western hypnosis, with the difference that the latter ‘does not require spells or odd statues’. That is why, he declares, modern people ‘should not discard talismans altogether’. The piece ends by urging the Chinese scientific community to use psychology and other sciences to study the efficacy of talismanic healing. ‘Who knows if one day we will not find out that talismans are even more effective and mysterious than hypnosis and could bring even more benefits to mankind?’^3^

Known in Chinese as *zhuyou* 祝由, talismanic healing refers to a range of ritual techniques in which written talismans and verbal spells are used as tools to prevent or heal disease. Although it was among the earliest medical disciplines recognised by imperial medical institutions—a status it enjoyed from the sixth to sixteenth centuries^4^—printed medical literature has persistently placed talismans and spells at the edge, if not entirely out of scholarly medicine. Until very recently, scholarship in English had maintained that talismanic healing has for millennia comprised mere ‘fringe activities’ of Chinese medical traditions.^5^ This teleological position, which intensified in the early twentieth century and has remained mainstream in medical histories in Chinese, also confines talismans and spells to a remote past, a time when the ‘Chinese did not understand the scientific principles of healing’.^6^ Seen as the antithesis of modernity and progress, talismanic healing is particularly marginalised in the medical historiography of the nineteenth and twentieth centuries, which tends to focus on the spread of Western biomedicine throughout China and the ‘invention’ of Traditional Chinese Medicine (TCM).^7^ To many, it is inconceivable that talismans and occult healing could survive, let alone thrive in the face of modern science and medicine. As the aforementioned anecdotes indicate—and to the discontentment of some—the popular press tells us a contrasting story about the place and value of occult arts in early twentieth-century China.

Talismanic healing constitutes what I call ‘the occult’ (*qi* 奇), a broad category that can be understood in both emic and etic terms. Emically, it includes a wide array of Chinese techniques—all of which were to some extent involved with healing and healthcare—such as astrology, divination, necromancy, geomancy, alchemy, spirit-writing and meditation. Typically classified as ‘arts and calculations’ (*shushu* 術數) in Chinese traditional histories and encyclopaedic works,^8^ these methods are based on the principle that ‘there are hidden processes and powers in the world that people can identify and tap in a variety of ways’.^9^ As forms of secret knowledge, they have been traditionally transmitted through master–disciple relationships, divine inspiration or the study of esoteric manuscripts. Secrecy is the sine qua non of their efficacy, with orality being paramount to the learning, practice and transmission of occult knowledge.^10^

Between the late nineteenth and early twentieth centuries, old Chinese terms like *qishu* 奇術, *mishu* 秘術 and *lingshu* 靈術 were redefined in Japan so as to include not only the traditional ‘arts and calculations’ but also Western magical tricks, mesmerism, hypnosis, Indian yoga and a wide range of new Japanese ‘mind-cure techniques’ (*reijutsu* 霊術).^11^ While this ‘new’ definition remains open-ended, I use ‘the occult’ in a rather etic sense. It refers not simply to the techniques per se but specially to those who endeavoured to ‘scientise’ them, to make them meaningful to a modern China. These I call ‘occultists’, educated men and women enthusiasts of the occult who attempted to make sense of their arts and beliefs ‘from the perspective of a disenchanted secular world’.^12^ As such, I do not focus on the large community of folk healers who were often the target of anti-superstition campaigns.^13^

From the late nineteenth to the mid-twentieth centuries, Shanghai emerged as the economic and cultural hub of China. The relative stability and political freedom enjoyed in the French Concession and International Settlement created what Wen-Hsin Yeh has called the ‘Shanghai splendour’.^14^ Thanks to Shanghai’s thriving publishing industry, an unprecedented influx of foreign political ideas spread from the city across the country alongside the dissemination of new technologies, new customs and new religious movements.^15^ While Chinese reformers agreed that to survive China should be modernised, their views on what ‘modern’ stood for varied. Likewise, whereas ‘superstition’ should be eliminated, to define what constituted ‘superstition’ was not an easy task. Some, like the co-founder of the Chinese Communist Party, Chen Duxiu 陳獨秀, believed that talismans, prayer, divination, spirit-writing and geomancy were ‘at odds with science’ and ‘belonged to the realm of fallacies’;^16^ but others, such as the psychical researcher Yu Pingke 余萍客, argued that ‘anti-science and superstitious’ were rather those who rejected the occult without having never investigated it.^17^ Historians have long hailed the views of those like Chen Duxiu as dominant in Republican China, silencing the existence of any dissonant voices. This article aims to give agency to those neglected figures whom, like Zhu Min and Yu Pingke, held that the occult could also heal the maladies and contribute to the modernisation of their country.

The late nineteenth century became a turning point in the history of the occult in China. Textbooks, pamphlets and manuals of occult arts mushroomed across the country, especially in Shanghai, which from the 1880s to the 1950s became the centre of China’s modern publishing industry.^18^ Schools of occult arts proliferated alongside tens of thousands of newspaper advertisements for astrologers, talismanic healers and spirit mediums offering their healing services and expounding the therapeutic benefits of their arts. Mass media did not only expand the public sphere wherein these ‘specialists of the occult’ had for long acted but also bolstered the market of occult arts to an extent never seen before. Driven by the hypnosis craze that swept across urban China, the popularisation of Western and Japanese spiritualist ideas led many to call for the disclosure of occult knowledge and its elevation as forms of modern, scientific knowledge.^19^

Recent scholarship has reflected upon the impact of new printing technologies on the production and consumption of Daoist and Buddhist texts, and how they transformed traditional religious ideas and practices in modern China.^20^ Inspired by Lauren Kassell’s concept of ‘economy of magic’,^21^ I will use the term ‘the market of the occult’ as an analytical framework through which to look at the production, distribution and consumption of goods and services related to the occult. While Kassell is concerned with the definitions and values of magic objects in seventeenth-century England, I shall focus on Chinese healing talismans as a means for exploring the formation of a market of occult arts in early twentieth-century China. From (i) the case study of Yu Zhefu 余哲夫, I will move to three other dimensions of Republican occultism: (ii) the publication of occult manuals for lay readers, (iii) the advertising of healing services and occult objects in mass media and (iv) the transmission of esoteric knowledge through textbooks and occultist schools. Chief among my goals is to argue that this market flourished thanks to the introduction of new printing technologies, the popularisation of mass media and the dissemination of Western and Japanese spiritualist ideas across urban China.

## Yu Zhefu and the *Compendium of Secret Talismans*

Compiled by the scholar Yu Zhefu, the *Compendium of Secret Talismans* is one of the earliest specialised texts of talismans produced in the form of a modern textbook.^22^ Printed by the Chenzhou Spiritual Society (*Chenzhou jingling xueshe* 辰州精靈學社) and distributed by the East Asia Press (*Dongya shuju* 東亞書局) and World Books (*Shijie shuju* 世界書局) in 1922, the *Compendium’*s first edition is divided into four volumes: ‘Healing Talismans of Zhuyou’ (*Zhuyou zhibing fuzhou* 祝由治病符咒), ‘Wondrous Talismans of Yuanguang’ (*Shenyan yuanguang fuzhou* 神驗圓光符咒), ‘Supreme Talismans’ (*Taishang wanling fuzhou* 太上萬靈符咒) and ‘Talismans for Nourishment’ (*Jingang jianshen fuzhou* 金剛健身符咒). While the *Healing Talismans of Zhuyou* records over a hundred talismans and spells used for healing, the *Talismans for Nourishment* is dedicated to talismans, spells and instructions for spiritual-cultivation and longevity. The volumes *Wondrous Talismans of Yuanguang* and *Supreme Talismans* are more diverse in content and include talismans for such purposes as the finding of lost or stolen objects, invocation of spirits, clairvoyance and exorcism. In total, the four-volume collection comprises almost four hundred talismans and spells employed for a wide variety of therapeutic needs. Later in 1924, Yu Zhefu sent to print a guide for the *Compendium*, a pamphlet titled *Research Methods for Talismans* (henceforth: ‘Research Methods’).^23^ We will return to this guide later.

Little is known about Yu Zhefu. Internal evidence from the *Compendium* suggests that he was born in Chenzhou, Hunan province, and that he had since early youth been interested in the study of ghosts and the occult.^24^ At least since the mid-Qing, historical records have heralded this city as the heart of talismanic culture in China, with the term ‘Chenzhou talismans’ (*Chenzhou fu* 辰州符) becoming a synonymous for healing talismans in posterior writings.^25^ A mid-nineteenth-century text traces the origin of talismanic healing to members of the Zhu clan, who had transmitted the practice in secrecy for generations.^26^ When passing by Chenzhou around the second century CE, the divine-physician Hua Tuo 華佗 entrusted a manuscript of talismans to a local friend. After Hua’s death in prison, the manuscript began to circulate among the inhabitants of Chenzhou, who then mastered the arts of talismanic healing.^27^ Interestingly, present-day Hunan belongs to the former kingdom of Chu (704–223 BCE), for millennia identified as the centre of shamanism in ancient China.^28^ Newspaper advertisements show that the overwhelming majority of talismanic healers offering their services across urban China highlighted their connection to Hunan.

Yu Zhefu, who in 1924 was between 70 and 80 years old, did not only come from Chenzhou but also belonged to the eleventh generation of a family of talismanic practitioners.^29^ According to a preface written by Hang Xinzhai 杭辛齋 (1869–1924) in 1923, Yu had dedicated the last 20 years of his life to the study of talismans and spells. Hang Xinzhai, a renowned political revolutionary, newspaper editor and specialist in divination, studied under Yu’s guidance in 1911. Zhang Taiyan 章太炎 (1868–1936) and Kang Youwei 康有為 (1858–1927), two prominent political reformers, also offered inscriptions and dedications to Yu’s book. All this demonstrates the affinity between reform-minded elites and occultists in the early decades of the Republic, a relationship traditional historiography has relentlessly tried to conceal.^30^ In the foreword of his *Compendium*, Yu states that for years he had convened study meetings with 12 comrades to research talismans and spells. Just as Hang Xinzhai, Zhang Taiyan and Kang Youwei, the foreword suggests that Yu was also a political reformer who fought to overthrow China’s last imperial dynasty: during the Xinhai Revolution (1911–12), his study group was temporarily dissolved and he had to flee to Japan to escape political persecution. It is likely that Yu’s connection to Western and Japanese spiritualist ideas began precisely during his years of exile in Japan^31^—a phenomenon not uncommon amongst his revolutionary comrades.^32^

Yu Zhefu’s authority as a specialist in talismans was singled out not only in the prefaces, inscriptions and dedications his book received, though. Publishers declared that his hitherto secret talismans, now made public through print, had for 11 generations been transmitted in secrecy—orally and by manuscripts—only among members of Yu’s ancestral lineage who lived in Chenzhou, the age-old centre of talismanic knowledge in China. But his book aimed not simply to reveal his ancestors’ esoteric writings; rather, it also promised to use simple language to explain their most arcane meanings to an audience beyond the limits of his clan and hometown. ‘Celestial mysteries have been leaked!’,^33^ ‘Unprecedent event!’,^34^ ‘Divine book has been published!’^35^: this powerful image of revealing secrets was constantly emphasised in the hundreds of advertisements for the *Compendium* published in Shanghai’s newspapers—and it soon became a staple motto among Chinese occultists at large.^36^

The importance of handwritten texts for talismanic learning should not be underestimated: rather than published in transcription form, the *Compendium* was for decades produced as a printed manuscript. The 1922, 1924, 1925 and 1935 editions of the *Compendium*, the only copies available to us today, were all produced through photolithography (*yingyin* 影印), a newly arrived technology publishers did not measure efforts to underline. In a series of advertisements issued in the 1920s, the East Asia Press and World Books announced, in large characters, that their edition of the *Compendium* was ‘purely a photocopy of Mr Yu’s original manuscript’, and assured that no changes were made to the printed version.^37^ This, combined with the fact that most of the specialised texts of talismanic healing that have survived to this day are manuscripts, reveal that the written word, even in its modern printed form, remained essential for the efficacy of talismans and spells.^38^ The use of manuscripts as a support for the transmission of talismanic knowledge and the practice of publishing talismanic texts as printed manuscripts both remain common in China today.^39^ In the case of Yu’s book, this strategy enjoyed a remarkable success: published for the first time in 1922, two years later the *Compendium* was already in its fifth edition. Advertisements for the book can be found all through the 1930s.

**Fig. 1.**
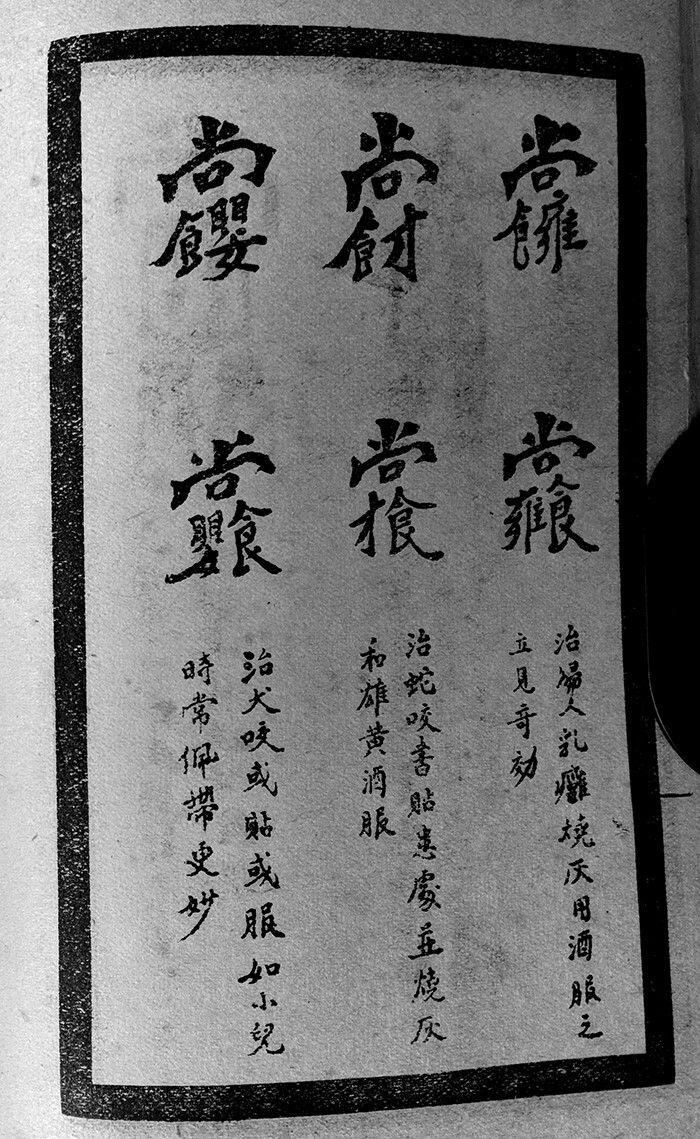
Example of three sets of three ‘secret characters’ for (from right to left) breast boils, snake bites and dog bites. The first set (right) must be burned to ashes and ingested with wine. The second set (middle) should be pasted onto the injured area, burned to ashes and then taken with realgar wine. The third set (left) can be pasted onto the injured area or ingested, and if the victim is a child, it is effective to carry it along with him or her for prevention. Extracted from Yu Zhefu, *Compendium of Secret Talismans*, 1922, II, 22

**Fig. 2.**
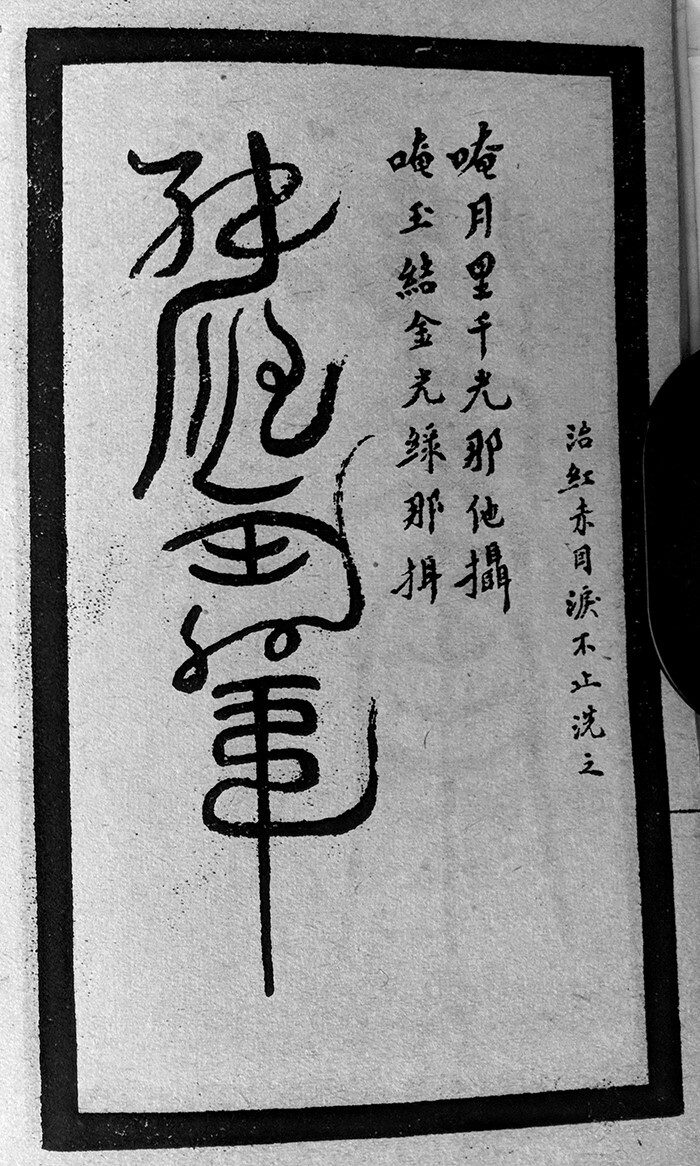
Example of an ‘elaborated talisman’ for red eyes. The talisman, which should be burned to ashes, mixed with water and used to wash the eyes, is accompanied by a verbal spell for activation. Extracted from Yu Zhefu, *Compendium of Secret Talismans*, 1922, II, 30

The success of Yu’s text must not be measured merely by its form as a printed manuscript. Following a broader trend among Republican occultists, the *Compendium* was organised in the style of a modern textbook for self-learners. Each volume begins by introducing the main contents therein contained, and then provides general instructions on how to write talismans and utter spells; when necessary, more detailed guidance is presented accordingly. For instance, the first pages of the volume *Healing Talismans of Zhuyou* are divided into ‘Notes’, ‘Ten Taboos’ and ‘Methods’, after which the text gives guidance on how to consecrate the paper, water, ink and other objects used for talismanic writing. The volume then lists dozens of ‘secret characters’ (*mizi* 秘字) used for healing, all of which are accompanied by herbal decoctions. The second part of the volume, on the other hand, is dedicated to ‘elaborated talismans’ (*huafu* 花符); each talisman should be activated by its respective verbal spell, burned into ashes and ingested with certain decoctions (Figures 1–2). The volume, however, does not teach about the sequence of strokes one must follow when writing talismans or how to set up the altar for the divine ancestor of this talismanic tradition. This lack of details prompted Yu to write a guide for his four-volume collection, which he completed in 1923: this was the process through which the *Research Methods* emerged as a separate publication.^40^

The guide is divided into 30 sections. After a historical account of talismans and spells in China, Yu Zhefu meticulously explains how to write ‘secret characters’ and ‘elaborated talismans’: just as when drawing a person, to design a talisman we should also begin with the head, move to the body and end up with the arms and legs.^41^ This allegory between talismans and the human body reveals that the former, after being properly written and activated, become ‘alive’ just as a sentient being. The *Research Methods* also lists several rules and taboos one should observe when creating talismans or uttering spells, as well as the various kinds of paper and ink that could be used depending on the type of effect desired. Yu classifies talismans into six categories, namely those to undo ill omens (*jie* 解), to suppress the evil (*zhen* 鎮), to heal diseases (*zhu* 祝), to invoke spirits (*qingxian* 請仙), for clairvoyance (*yuanguang* 圓光) and spirit-writing (*fuji* 扶乩). Talismans written in secrecy, he assures, are still the most effective. In the section ‘Researching Zhuyou’ (*Zhuyou yanjiu* 祝由研究), Yu explains how to set up an altar to the Perfected Man of Great Unity (*Taiyi zhenren* 太乙真人), the ancestor of his talismanic tradition and the god to whom the healer should beseech through talismans and spells.

Yu Zhefu’s pursuit for transforming talismans and spells into a modern field of study was later materialised with the formation of the Research Institute of Chenzhou Talismans (*Chenzhou fushu yanjiuhui* 辰州符術研究會) in the early 1920s, a group dedicated to the study of talismanic rituals. Not much is known about the Institute. A series of advertisements from 1924 state that Yu established it following the success of his *Compendium*. Those who bought the book could automatically join the Institute as official research members (*yanjiuzhe huiyuan* 研究者會員) and clarify any doubts face-to-face or via correspondence.^42^ Distance learning, which challenged the traditional means through which to learn esoteric knowledge, became a recurrent practice among Chinese occultists. Members of the Institute could also enjoy discounts in future publications and have exclusive access to a textbook Yu specially designed for his new students.^43^

While textual evidence does not permit us to verify how long the Institute lasted, we do know that Yu Zhefu was the director of another research group, the Chenzhou Spiritual Society, dedicated to the study and trade of occult manuals and talismans. The Society worked closely with Chinese spiritualist groups, publishers and bookstores, including the Academy of Occult Arts (*Qishu zhuanxiuguan* 奇術專修館), a school committed to the study and training of students interested in the occult. In an advertisement on 4 June 1923, Yu and the Academy’s director, Liu Shenfeng 劉神鳳, announced the selling of 55 talismans created by a 96 years-old Daoist named Hefa daoren 鶴髮道人.^44^ In order to raise funds for the restoration of his temple in Sichuan, Hefa daoren had previously issued several newspaper advertisements selling his talismans. In 1922, for example, the Society of Spiritualism (*Lingxue hui*靈學會) declared it was helping the old Daoist raise funds through the trade of his ‘powerful healing talismans’.^45^ Two years later, the Chenzhou Spiritual Society and the East Asia Press co-advertised the selling of Yu’s manual and a couple of talismans written by the Celestial Master, a key figure within Chinese Daoism.^46^

The vocabulary Yu Zhefu employs in his texts deserves due attention. Whereas words like *xuexi* 學習 (to study), *yanjiu* 研究 (to research) and *zhengming* 證明 (demonstrate empirically) are seldom if ever found in talismanic manuscripts from late imperial China, these terms are all pervasive throughout the *Compendium* and its advertisements.^47^ The title of his guide, *Research Methods for Talismans*, and its various entries on *zhuyou* (healing talismans), *yuanguang* (clairvoyance) and *fuji* (spirit-writing)—all of which formed with the suffix -*yanjiu* (to research)—demonstrates Yu’s intention in transforming hitherto secret knowledge into a public field of study.^48^ This is again emphasised in the preface of his *Research Methods*. Here, he criticises that talismans and spells had traditionally been ‘embraced in mysticism’ and ‘transmitted only from fathers to sons but not to sons-in-law’—that is, only among blood relatives.^49^ His manual, however, tried to overcome all this: it was public, modern and scientific.

The popularisation of Western and Japanese psychology, spiritualism and psychical research in early twentieth-century China prompted the widespread belief that talismans and spells were not superstitious practices—as certain critics had denounced—but instead powerful psychic techniques used for spiritual healing. Their efficacy could now be explained on the same basis as mesmerism, hypnosis and other Western psychic cures.^50^ Such claims can be found in advertisements for the *Compendium* and various other talismanic manuals, many of which went as far as to declare Chinese talismans to be superior to Western hypnosis in terms of therapeutics.^51^ While praising talismans and spells as powerful tools to treat ‘diseases of the mind’, Yu promoted that the efficacy of *yuanguang*, a traditional healing technique which combined principles of divination and clairvoyance, lay entirely in ‘psychological factors’. This exemplifies what Wouter J. Hanegraaff has called the ‘psychologisation of the sacred’,^52^ a tendency that flourished in China following the dissemination of spiritualist ideas.

Alongside healing talismans, Chinese spiritualists were fascinated by the study of *yuanguang* as a means for them to set up a bridge between China, Japan and the West in matters of paranormal studies. Divination and clairvoyance were among the chief phenomena which captivated the minds of spiritualists and psychical researchers at that time.^53^ In China, many now celebrated the wide array of Chinese occult arts and the enduring fascination they exerted over the population at large not as obstacles to modernisation but rather as an essential contribution to the development of modern science and medicine, a contribution that transnational psychical research was engaged to materialise.^54^ It is certainly not a coincidence that guides for *zhuyou* and *yuanguang* talismans proliferated side by side in Republican China, being often sold as parts of larger collections of ‘occult’ (*qishu*) and ‘spiritual’ (*shenshu*) healing arts. It is noteworthy that *qishu* and *shenshu* were concepts in vogue among early twentieth-century Japanese spiritualists and later adopted by their Chinese fellows to indicate ‘mind-cure techniques’.^55^

Empirical investigation and democratisation of knowledge were among the hallmarks of the transnational spiritualist movement that washed over the world in fin de siècle.^56^ The term ‘exotericism’ (*gongkai zhuyi* 公開主義), coined as a contrast to ‘esotericism’ (*shenmi zhuyi* 神秘主義), became a powerful motto among early twentieth-century Chinese spiritualists. The Society of Spiritualism, for instance, had for decades not simply conducted ‘scientific séances’ as a means for understanding the spirit world but also published the entire séances’ records in its periodicals, a practice utterly uncommon in traditional altars of spirit-writing.^57^ The Chinese Institute of Mentalism (*Zhongguo xinling yanjiuhui* 中國心靈研究會), on the other hand, published hundreds of manuals and textbooks of hypnosis, clairvoyance and mind-cure techniques, training thousands of Chinese students from China and overseas. The Institute also played a key role advocating talismanic rituals as the Chinese version of Western hypnosis.^58^ Apart from talismans, hypnosis and spirit-writing, the Academy of Occult Arts regularly offered courses of magic, geomancy and a number of traditional divination techniques, including fortune-telling, palmistry and physiognomy.

The trend for making secret knowledge public materialised in the remarkable number of public performances Chinese occultists conducted between the 1910s and 1930s. While these performances were more common among hypnotists and magicians, the practice spread to other specialists of the occult as well, including talismanic healers. Advertisements from 1924 and 1925 report that Yu Zhefu conducted several public demonstrations in a temple in southern Shanghai. The performance carried out on 22 June 1924, a newspaper states, lasted for six hours and was watched by over a thousand people, including members of the Spiritualist Society (*Xinling xueshehui* 心靈學社會) and Chinese Institute of Occultism (*Zhongguo shenmi zhexue* 中國神秘哲學). The announcement then describes the address, profession and name of 10 individuals who participated in Yu’s performance, including Zhang Ziyang 章子祥, an entrepreneur whose inflammation was cured by talismans within 14 minutes.^59^ Those who did not attend the performance, a publisher persuasively suggested, could instead buy the *Compendium*: the talismans therein recorded promised to be as effective as if one had received teachings directly from Yu’s mouth.^60^

The use of first-hand witnesses as a means for demonstrating the efficacy of talismans and spells is equally found in dozens of testimonials of individuals who were healed after applying the instructions published in the *Compendium*.^61^ On 9 July 1922, a letter from Zhang Shaozhen 張紹楨, an officer from the Bank of China in Hong Kong, praises the publication of the manual after describing how he used a divination technique to find his lost watch. Two years later, the East Asia Press and World Books published the testimonials—together with the address, profession and name—of over 20 people who successfully applied Yu’s talismans. Over the 1920s, the personal experience of dozens of individuals was published as part of larger advertisements for the *Compendium*. Individual testimonials served to counterbalance the fact that even as a printed (= public) text, Yu’s manual remained remarkably effective: oral transmission, divine inspiration and the study of esoteric manuscripts were no longer necessary as long as one followed the instructions recorded in printed word.

## Publishing Secrets

The publication of such talismanic manuals as Yu Zhefu’s *Compendium* was part of a larger market formed around a growing interest in occult matters. Driven by the popularisation of mass media, cheap print and spiritualist ideas, this market boomed in the first half of the twentieth century to unprecedented heights. Alongside handbooks of talismans and spells, a plethora of almanacs and guides of astrology, divination, hypnosis, Indian yoga, geomancy, meditation and necromancy integrated the bookselling market of Republican China—as publishers’ catalogues, newspapers and in-book advertisements convincingly attest.^62^

While the publication of these texts might have been partly motivated by the very capitalist nature of Shanghai’s publishing houses,^63^ we should not overlook the peculiarities of occult knowledge itself: oral transmission and divine inspiration, often supported by the materiality of esoteric manuscripts, were the sine qua non of occult knowledge’s efficacy. The publishing of secrets in the form of cheap ‘know-how’ texts did not simply open up new channels for accessing esoteric knowledge but also assured that the techniques transmitted through the printed word could be as effective as those passed down by traditional means. Simply put, the publication of occult texts would make no sense if readers did not accept that printed manuals were also legitimate—and, in turn, effective—channels for transmitting secret knowledge. The China and West Press (*Zhongxi shuju*中西書局; henceforth: ‘Press’) thoroughly illustrates the engagement of Shanghai’s publishers in the formation of this market of the occult.

The Press, a midsize publishing house founded by Wu Lugong 吳盧公 in 1917, was specialised in the trade of cheap books, pamphlets and reprints. Primers and household manuals, along with novels and medical texts, constituted the core of its bookselling business.^64^ From the early 1920s to its closure in the 1940s, the Press invested massively in the publication of occult texts as well. While in 1926 it announced, in a modest advertisement, the publication of only nine titles,^65^ three years later the number jumped to twenty-one and occupied over one-third of a newspaper’s page (Table 1).^66^ This number is reached if we count the *Collection of Occult Arts* as a single book: its 81 volumes, however, contained 13 different titles on geomancy, divination, physiognomy, necromancy, hypnosis, astrology and talismans. Compiled by the founder of the Academy of Occult Arts (AOA), Liu Shenfeng, the *Collection* ranked among the Press’ bestsellers, being advertised in all Shanghai’s major newspapers for nearly a decade. From a title catalogue published in 1927, we are informed that by then the Press was selling well above 80 manuals on a plethora of Chinese, Western and Japanese occult arts.^67^ Many of the Press’ manuals were reprinted by, of reprints from, other contemporary publishers.

**Table 1.** List of the occult texts the China and West Press highlighted in the *Xinwenbao*, 29 November 1929.

Title in Chinese	Title in English	Compiler	Main Content
白光電球奇術	The Occult Arts of Radio-Hypnosis	AOA	Hypnosis
奇門遁甲秘笈	Esoteric Arts of Qimen Dunjia Divination	AOA	Astrology and divination
奇術叢書	Collection of Occult Arts	AOA	Various occult arts
算命講義大全	Compendium of Fortune-Telling	Bogu shulou	Fortune-telling
驚人相術奇術鑑人術	Occult Arts of Physiognomy	Feng Yunzi	Physiognomy
陰陽地理風水講義	Lectures on Yin-Yang, Geography, and Fengshui	Foyin shuju	Geomancy
圓光真傳秘訣	Secret Transmission of Yuanguang	Foyin shuju	Clairvoyance and divination
扶乩真傳秘訣	Secret Transmission of Spirit-Writing	Guo Ren	Necromancy
六壬學講義書六壬鑰	Lectures on Liuren Divination	Jiang Wentian	Astrology and divination
辰州符咒大全	Compendium of Chenzhou Talismans	Mojing shuwu	Talismans
關亡召鬼秘術	Secret Arts of Necromancy	Mojing shuwu	Necromancy and spirit-photography
祈夢秘書	Secret Book of Dream Interpretation	Shi Shilun	Dream creation
妖怪攝精煉形奇談	Fantastic Tales of Monsters and Spirits	Shi Zhulu	Necromancy, exorcism, and ghost-catching
神傳護身術	Divine Arts of Self-Cultivation	Sohaku Nakazawa	Martial arts, breathing and meditation
催眠術講義	Lectures on Hypnosis	Wei Quanzi	Hypnosis
祝由科治病奇術	Secret Book of Healing Talismans	Xu Jinghui	Talismans

Promoted under the category of *qishu*, or ‘occult arts’, the manuals were designed as modern textbooks for general readers who might not have prior tacit knowledge in the occult, ‘starting with the simple and then concluding with the complex’.^68^ Sold for a low price, written in vernacular Chinese and printed in transcription form—except for talismans, which are still printed as photocopies of manuscripts—each manual offers detailed instructions for use and promises to reveal secrets that were hardly if ever disclosed in traditional occult texts. The *Secret Book of Healing Talismans* (henceforth: ‘Secret Book’), which has survived in dozens of Republican editions and is still in print today,^69^ epitomises how the Press’s manuals differ from pre-twentieth century occult texts.

The *Secret Book* is an expanded and commented version of a popular talismanic manual compiled in the mid-nineteenth century, the *Yellow Thearch’s Stele of the Thirteen Disciplines of Zhuyou* (henceforth: ‘Stele of Zhuyou’).^70^ Although the *Stele of Zhuyou* is more detailed compared to talismanic manuscripts, its level of detail is not a match for the *Secret Book*. First, all the talismans contained in the *Secret Book* are simple and composed of well-defined characters, while in the *Stele of Zhuyou*, over half of them are elaborated talismans, which are far more difficult to learn, memorise and write (Figures 1–2). Secondly, whereas the *Stele of Zhuyou* contains only one half-page introductory text—which merely describes the history of the text’s transmission—the *Secret Book* has nothing less than nine spreading over 16 pages. It includes entries on the origins of talismanic healing, the rules and taboos both healer and patient should observe, methods for writing talismans and uttering spells, how to assess the health condition of a patient and set up an altar for talismanic rituals. Thirdly, in the *Stele of Zhuyou*, for example, an elaborated talisman to invoke the God of Incantations is only followed by three spells and two proper names, Ying Daozhou 應道周 and Yuan Zhi 袁智, leaving the reader with absolutely no idea about what all this is supposed to mean.^71^ In the *Secret Book*, by contrast, the elaborated talisman and two spells are removed, and we learn that the two proper names refer to the divine names of the God of Incantations, whose healing powers should be invoked through a verbal spell and his written names.^72^ With regard to instructions, talismanic manuscripts are even more modest than the *Stele of Zhuyou*, indicating that their purpose was to conceal, rather than reveal occult knowledge.^73^

Hypnosis became a driving force for the rediscovery of traditional occult arts in Republican China. Celebrated as a scientifically proven psychic technique (*lingshu*), hypnosis promised to heal all sorts of physical and psychological diseases. And it promised much more. Renowned Western and Japanese psychical researchers declared that during the hypnotic state highly sensitive persons could foresee the future, perform astral projection, diagnose the illness of people not physically present and even talk to the dead.^74^ Thousands of studies on the wondrous effects of hypnosis began to be translated into Chinese and published in the popular press. A hypnosis craze swept across urban China, prompting the formation of hundreds of spiritualist and occultist groups dedicated to the study of hypnosis and the occult.^75^ And the more Chinese elites became familiar with Western and Japanese hypnotic techniques, the easier it was for them to identify similarities between these and Chinese occult arts. In the preface for Guo Renlin’s 郭仁林 book *Treatise on Ghosts*, for instance, the scholar Ouyang Zhongshou 歐陽仲濤, an active member of the Society of Spiritualism, proclaims that prayer, divination, astrology, bone-reading, necromancy and spirit-writing are ‘all hypnotic techniques used for communication with the world of the spirits’.^76^ The thousands of writers and publishers that echoed Ouyang’s words indicate that he was by no means a dissonant voice within the broader community of Chinese occultists.

Within this context, the market of occult texts gained a new lease of life under the auspices of the spiritualist movement. Not surprising, the China and West Press was located on the same street as the headquarters of the Society of Spiritualism.^77^ The Press’s founder, Wu Lugong, began his publishing career in the World Books—which had not only distributed and promoted the *Compendium* for years but also invited Yu Zhefu to conduct public performances in Shanghai in 1924 and 1925. A closer look at manuals and advertisements shows the impact ideas pushed forward by Chinese spiritualist groups had on the Press’ publishing activities.

In a title catalogue published in 1927, the Press spent most of its pages introducing the occult texts it had long advertised in commercial newspapers. Through a combination of texts and images, individual entries showcase the specificities of each manual. Words like ‘psychical research’ (*jingshenxue*), ‘spiritualism’ (*xinlingxue*), ‘psychology’ (*xinlixue*), ‘science’ (*kexue*) and ‘to demonstrate empirically’ (*zhengming*), all pervasive in spiritualist works, permeate the whole catalogue. In an entry for *yuanguang*, for instance, the text begins by claiming that this is a ‘traditional art of our Country’ which ‘essentially comprises a branch of psychic research’. But the manuscript from which the manual was produced, the texts assures, ‘had for centuries been passed down in secrecy’.^78^ Drawing from studies conducted among psychical researchers in the West and Japan, since the 1910s Chinese spiritualists began to hail traditional *yuanguang* as a technique of clairvoyance and telepathy. Suddenly, specialised texts of *yuanguang*, which are seldom mentioned in private collections before the twentieth century, mushroomed in Republican China, with hundreds of journal articles debating its marvellous effects. Reports of individuals who used *yuanguang* to diagnose the illness of people not physically present, to exorcise evil spirits or to harness the healing power of divine beings all flourished in the Chinese press.^79^

The catalogue also dedicates several pages to introducing the latest psychic methods coming from the West. The Knowles Radio Hypnotic-Crystal, a device invented by the world-renowned occultist Elmer E. Knowles (1861–1959) for hypnotherapy and attention focus,^80^ was announced together with *The Occult Arts of Radio-Hypnosis* (*Baiguang dianqiu qishu*), an ‘acclaimed manual that has already been translated into dozens of Western languages’.^81^ Praising it as an ‘innovative science popular among intellectuals and used as a powerful pathway by electrical engineers, spiritualists, philosophers, physicians and psychologists worldwide’, the Press urged readers to personally experience what radio-hypnosis had to offer in terms of mind-reading, healing, clairvoyance and necromancy.^82^

**Fig. 3.**
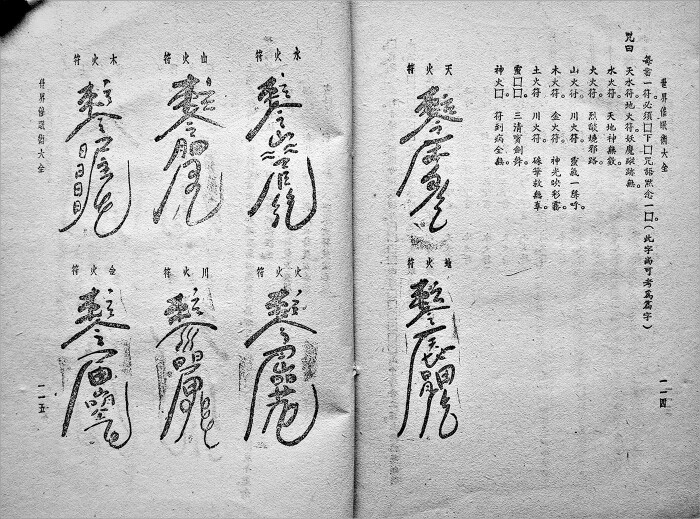
Eight ‘elaborated talismans’ for inducing hypnosis. Extracted from Huang Feng, *Collection of Global Hypnosis*, 1940, 24–25

The Chinese arts of talismanic healing, occultists declared, worked on the same basis as hypnosis.^83^ Since its invention in the nineteenth century, however, nowhere a consensus has existed about how and why hypnosis exactly works.^84^ While some related its principles of efficacy to the brain, others found more sense in psychological and spiritual explanations.^85^ The claim that talismanic rituals worked as a form of psychotherapy (*jingliaoxue* 精療學) soon became mainstream among Chinese occultist circles. An article published in 1937, for instance, argues that the efficacy of talismanic rituals lies in that the healer gives suggestions to the sick by setting up altars, burning incense and writing talismans.^86^ The ancients, others claimed, vested talismanic rituals in a religious guise not only because they did not have the scientific means to study it properly but also because they realised that treatments were always more effective when the sick’s faith increased.^87^ Although not denying the power of the gods altogether, several occultists praised the ‘expectation effect’ (*yuqi zuoyong* 預期作用), this ‘remarkable scientific discovery’, as the real phenomenon talismans and spells could help create.^88^ Consequently, the most popular guides of hypnosis published in Republican China included discussions on talismanic healing. Whereas Wang Yang 汪洋’s manual *Chinese & Foreign Hypnotism* discusses at length the relevance of prayers, spells and talismans as psychic cures,^89^ Huang Feng 黃楓’s *Collection of Global Hypnosis* records dozens of Chinese talismans for inducing the hypnotic state (Figure 3).^90^

Not only talismans and *yuanguang* attracted the attention of Chinese spiritualists. While the *Arts of Physiognomy* claimed to combine the best from Chinese, Japanese and Western arts of physiognomy, all ‘scientifically proven’,^91^ the *Fantastic Tales of Monsters and Spirits* contained techniques to invoke, exorcise and manipulate spiritual beings.^92^ Mediumship was the hallmark of the spiritualist movement worldwide, and combined with the Chinese longstanding attraction for the spirit world, it generated a rich literature on the academic and practical dimensions of this subject. The revival of spirit-writing altars in urban areas might be linked with the popularisation of Western spiritualism across China too.^93^ Declaring itself as a must among such altars, the *Secret Transmission of Spirit-Writing* promised to reveal all the secrets for spirit-communication. The *Secret Arts of Necromancy* went even further, dedicating several chapters to the ‘scientific arts of necromancy and spirit-photography’. In fact, the manual’s alternative name, ‘Proven Studies of the Spirit’ (*Shiyan linghunxue* 實驗靈魂學), was an allusion to the field of psychical research, often translated in Chinese as *linghunxue* 靈魂學. The fascination occult healing and the spirit world exerted over the burgeoning community of Chinese spiritualists—many of whom were also concerned with the modernisation of China—indicates that these groups constituted the main target readership of the Press’s manuals.

## 4. Advertising the Occult

In 1998, Owen Davies highlighted the potential of newspapers to enhance our understanding of witchcraft and magic in modern England. While the belief in traditional witchcraft waned during the early twentieth century, Davies argued, newspapers reveal that the same period witnessed a growing acceptance of divination and astrology, a ‘harsh reality’ for the enthusiasts of post-enlightenment positivism. Meanwhile, newspapers also created a space wherein the cunning-folk and fortune-tellers could interact with the larger public without the intermediation of British intellectuals—many of whom were averse to ‘old superstitions’.^94^ In Republican China, mass media also played a fundamental role in the formation of the market of the occult. Newspapers served a medium through which to advertise the trade of occult objects, the therapeutic service of occultists and the activities of occultist schools involved in the transmission of esoteric knowledge.

As a bestseller of its day, Yu Zhefu’s *Compendium* also served as a channel for publishers to sell ready-made talismans produced in accordance with the manual’s instructions. As part of an advertisement for the *Compendium*, in 1922, the East Asia Press and the Chenzhou Spiritual Society co-announced in the *Shanghai News* the selling of healing talismans directed to all sorts of internal and external illnesses, including bones stuck in the throat and *nüe* 瘧, an epidemic disease whose cause was often attributed to demons. Those interested should send an order to the East Asia Press with the kind and quantity of talismans desired and could ‘test them on the spot before purchase’. Each talisman, created by Yu himself, was being sold on consignment in the publisher’s headquarters in Shanghai for the price of one silver dollar and five cents.^95^

Not all talismans were meant to be sold, though. Newspapers reveal that talismans were commonly sent as gifts to customers of certain products. On 10 June 1922, the *Eastern Times* notified readers that in that week it would send to everyone a talisman for mad dog bites.^96^ Three years later, the World Books announced that those who buy their books would be gifted a photo of the Panchen Lama, which served as a potent amulet to ‘expel the evil and attract good fortune’.^97^ In 1929, a series of advertisements for a brand of pills for improving parturition declared that all customers would receive a divine talisman for safe delivery as a goodwill gesture.^98^ And in July of the same year, the Society of Spiritualism, which had for decades been involved with the study, production and distribution of talismans, announced that a spirit-photography of, and a talisman written by the Immortal of Purple Clouds (*Zixia zhenren* 紫霞眞人) would accompany their new edition of the ‘Records of Human-Spirit Communication’ (*Rengui tongling lu* 人鬼通靈錄).^99^

The publication and distribution of medical and religious books for free as a means for individuals to accumulate good karma were pervasive in traditional China.^100^ The popularisation of mass media in the twentieth century did not only invigorate this practice but expanded the readership (and consequently, the sponsor’s good karma) to unprecedented levels. In 1922, a man whose mother was sick announced that he would sponsor the publication of 500 copies of Yu’s *Compendium* at half of the original price.^101^ On 6 February 1895, another individual offered detailed instructions on how to create a simple yet ‘effective divine talisman for a difficult labour’ that anyone could use in an emergency.^102^ And stating that the summer of 1918 had been excessively hot and that this would inevitably cause epidemics in autumn, on August of that year an office in Shanghai announced the distribution for free of ‘divine talismans against demonic epidemics’, which could be delivered by mail or collected in person.^103^

In a series of advertisements issued in the *Shanghai News* between May and June 1915, a patient named Ji Zhonglu 季仲魯 used a talisman to advertise the medical activities of the Ren’an yiju 仁安醫局, a hospital in Shanghai specialised in the treatment of venereal diseases. Ji Zhonglu, the announcement begins, had long been afflicted by bubo. After trying all kinds of conventional remedies in vain, Ji begged his relative, the medium Liu Renfu 柳任甫, to ask the spirits for a cure. A spirit presented them with a talisman yet provided no instructions on how to use it. Not knowing how to proceed, Liu asked the spirit for clarification, to which the spirit responded:

The reason master Liu does not understand how to use the talisman is that it should neither be ingested nor pasted. This talisman rather comprises the four characters ‘仁’ ‘安’ ‘醫’ ‘局’. Divine physicians are not as good as human physicians! Rush to the Ren’an Hospital and you shall be healed in half a month.

This was not a conventional talisman—which the sick should otherwise ingest or paste somewhere—but a ‘divine admonition’, written in talismanic form, urging Ji to visit the Ren’an Hospital. Healed after following the spirit’s instructions, Ji printed the talisman as proof of what happened and exhorted those suffering from the same disease to visit the hospital immediately.^104^ Here, the authority of the spirits, combined with the material support provided by print, transformed the talisman from a secret writing into a modern form of advertisement.

Talismans printed in newspapers also served to promote talismanic manuals and demonstrate their efficacy to prospective customers. In 1924, a large advertisement for the *Compendium* is composed of two talismans, one for *nüe*-disease and another for cough. At the top of the talismans, a passage explains their effects and instructions for use: each talisman should be activated by its respective verbal spells in the early morning. Below the talismans, though, a text written in tiny letters states that the spells are contained inside the book—that is, to know the activation spells, the person should purchase the entire collection.^105^ This practice spread to such an extent that in 1935, the Bright Spring Books (*Chunming shudian* 春明書店) published, as part of their advertisement for the *Compendium*, a talisman for warding off *nüe-*demons. This time, the bookstore provided a whole extract from Yu’s text, which explains what *nüe-*demons are, how to create the talisman and the spell to activate it.^106^ The act of printing talismans in newspapers and encouraging readers to personally appraise their efficacy became an important marketing strategy in Republican China.

Although the circulation of talismans and other occult artefacts can be easily detected in newspapers, it was the activities of Chinese occultists which truly dominated the press. Advertisements of talismanic healers offering their healing services exploded alongside those of individuals seeking, or recording their personal experience with, talismanic treatments. As a specialist in talismans, divination and astrology, the case study of Yang Haogu 楊好古 is exemplary. Little is known about his life, despite that he came from Chenzhou and had for years established a healthcare market in Shanghai.^107^ From 1914 to 1932, over three thousand advertisements issued by Yang or his numerous clients were published across Shanghai’s newspapers. Self-identified as a ‘talismanic-physician’ (*zhuyouke yishi* 祝由科醫士) from a lineage of specialists in Chenzhou talismans, Yang advertised treatments for all sorts of diseases that conventional medicine was unable to treat. ‘Contrary to the dangerous drugs imposed by Western and Chinese doctors’, he declared, ‘I heal the sick by employing the psychotherapy technique of talismans.’^108^ His acquaintance with the spiritualist movement is beyond doubt.

Using talismans alone or in combination with massage and acupuncture, Yang Haogu promised to treat cough, blood disorders, bone injuries and especially illnesses of children, women and those provoked by evil spirits.^109^ Since 1916, he had also begun to announce talismanic cures for diseases caused by bacteria and germs,^110^ along with services of divination, astrology and clairvoyance.^111^ After attending for years at several hostels in Shanghai, he eventually established a private altar in a central area of the International Settlement.^112^ Later in 1927, his success as a talismanic healer led he and his wife to open a small-sized clinic in a prime location at central Shanghai. While he administered treatments with astrology, talismans and divination, his wife, a spirit-medium, took care of the gynaecology department.^113^ As for 1932, Yu Hualong 俞華龍, another renowned talismanic healer working in Shanghai, informed a local tabloid that occult arts made Yang so wealthy that he decided to leave the city and retire to the countryside.^114^

Yang Haogu did not restrict his activities to Shanghai but travelled throughout China, including the large centres of Hankou, Beijing and Tianjin. Individuals from Guangdong, Yangzhou and Tianjin also praised his wondrous healing skills, publishing thanking letters to him in all major newspapers. An idea of his clientele can be drawn from a series of advertisements published in 1926. Here, we are informed that in a trip to the north, Yang successfully treated a number of aristocratic families, including the daughters-in-law of the warlord Zhang Yuting 張雨亭 (1875–1928) and the scholar Yuan Hanyun 袁寒云 (1889–1931), a leading writer of the influential tabloid *The Crystal* (*Jingbao* 晶报). His two most distinguished clients were Li Yuanhong 黎元洪 (1864–1928), an enthusiast of Chinese spiritualism who assumed the presidency of the Republic of China twice, and the former emperor Puyi 溥儀 (1906–1967), both who enjoyed Yang’s healing and astrological services.^115^

Yang Haogu was by no means alone in China’s market of healing talismans. Quantitative analysis shows that from the 1880s to the late 1930s, over 20,000 advertisements for talismanic healers were published only in the *Shanghai News*, *Shanghai Daily* and *Eastern Times*, with a boom between the 1920s and 1930s.^116^ This number does not include advertisements for geomancers, hypnotists and the whole plethora of specialists in occult arts other than talismans. Not surprisingly, that period marked the peak of the Chinese spiritualist movement: fostered by a prosperous print culture, dozens of spiritualist groups devoted to the study of the occult were running throughout urban China by the mid-1920s.

## 5. Occultist Schools and the Transmission of Esoteric Knowledge

Similar to the German occult movement Corinna Treitel investigated, modern Chinese occultism was ‘very much a public enterprise’.^117^ Occultists engaged in heated debates on the pages of China’s wide-circulation newspapers, organised public demonstrations, published inexpensive editions of occult texts, advertised healing services and even welcomed prospective students to apply for admission to occult courses designed for laypeople. Indeed, the transmission of esoteric knowledge was among their main concerns. The Academy of Occult Arts, which worked closely with Yu Zhefu’s Research Institute of Chenzhou Talismans, is a case in point. The Academy was founded in the early 1920s by Liu Shenfeng, an occultist, former emissary at the Ministry of Rites and former member of the Imperial Board of Astronomy, a man who ‘has long enjoyed researching ancient occult arts’.^118^ It worked as a publishing house for occult manuals, a store for occult objects and a school for distance learning.

In 1923, a series of advertisements for prospective students lamented that ‘while such occult arts as astrology, geomancy and divination have existed in our country for millennia, true books have seldom been disclosed to the public’. Folk masters, on the other hand, ‘often use their arts to deceive others’, increasing people’s suspicion toward occult matters. ‘But true books do exist’. After the fall of the Qing dynasty, the advertisement continues, ‘Mr Shenfeng spent years travelling across the four seas in search of masters of extraordinary powers’. He ‘learned all the secrets and collected all proven texts’ and is now devoted to ‘propagate occult arts and identify past mistakes’. His Academy focused on correspondence courses of divination, physiognomy, fortune-telling, geomancy, *yuanguang*, talismans, as well as three ‘new kinds of occult arts’: a ‘divine technique’ invited by a Japanese doctor surnamed Matsuda 松田, hypnosis and magic.^119^ Asking for one yuan as tuition fee, the Academy promised to accept up to 50 students per term, whom upon the completion of their studies—expected to happen in a month—would then receive a printed diploma: all this enjoying the comfort of one’s home.^120^ By late 1926, the Academy boasted having already taught over a thousand students.^121^

The Academy integrated a far broader education system for learning occult knowledge in Republican China. Alongside the Society of Spiritualism and the Chinese Institute of Mentalism, other major spiritualist and occultist groups such as the Chinese Hypnotism Association (*Zhongguo jingshen yanjiuhui* 中國精神研究會), the Heavenly Society of Spiritualism (*Shenzhou lingxue zonghui* 神州靈學總會) and the Chinese School of Abnormal Psychology (*Zhonghua biantai xinlixue* 中華變態心理學) all promoted the study and practice of occult arts as forms of modern, scientific knowledge. The dozens of new religious movements that washed over the first half of the last century also helped propagate occult healing across social borders.^122^ Distance learning, combined with the proliferation of schools and textbooks directed to lay readers, did not only try to turn upside down the traditional model of apprenticeship but also reinforced the boundaries between ‘modern/literate’ and ‘traditional/folk’ practitioners. The case study of the fengshui master Tan Yangwu 談養吾 (1880–c. 1950s) illuminates this.

Tan Yangwu, a member of the prestigious Mystic Void (*Xuankong* 玄空) lineage of geomancy, learned this art from Yang Jiuru 楊九如, a relative of the lineage’s founder, Zhang Zhongshan 章仲山. What began as a conventional means for learning esoteric knowledge soon became a model that challenged the very roots of occult traditions. After spending years learning geomancy, Tan began to feel upset about how this art had traditionally been taught. The Mystic Void, he complained, was so involved in secrecy that even disciples would not learn everything their masters knew. This caused its members ‘to care only for the study of old writings’ and close themselves to the innovations of the modern age. Overlooking the existence of other geomantic lineages, followers engaged in the ‘fabrication and proliferation of mistakes’. Echoing the voice of Yu Zhefu and his fellow occultists, Tan lamented that over time secrecy had transformed the Mystic Void into an ‘array of heterodoxies’.^123^

In order to restore the Mystic Void’s past glory and make its knowledge useful to the modern world, Tan founded the Three Principles Institute of Occult Arts (*Sanyuan qishu yanjiushe* 三元奇術研究社) in Shanghai in 1922. The Institute was devoted to the practice and teaching of fengshui, as well as the publication of geomantic manuscripts transmitted within his family. In the early 1920s, Tan published the *Three Principles Geographic Guide of the Great Mystic Void*, his magnum opus, which served as a textbook for new students in his Institute. For the price of two yuan per month, the Institute promised to ‘reveal all the most arcane secrets of geomancy to anyone interested’.^124^ Indeed, the Institute had students from all over China, including Hong Kong.^125^ Between the 1930s and early 1940s, Tan published articles on geomancy and science to general readers, conducted public performances, cast geomantic charts for private companies, selected auspicious places to build the tombs of Chinese aristocrats and even had his own daily radio programme.^126^

Intellectuals, Tan lamented, avoided the topic of fengshui by blindly condemning it as superstition, while scientists did not know the proper means to study it. As an occultist acquainted with modern science, Tan not only posed himself as an intermediate between these two groups but also as a contrast to folk geomancers, whom he despised as chief vectors of superstition. Following the trend among Chinese occultists, he championed the scientific study of occult arts as the best way to eradicate superstition from Chinese society. Throughout his books, Tan sought incessantly to explain the efficacy of traditional fengshui vis-a-vis the modern Western disciplines of geography, physics, astronomy, mathematics and meteorology. And he was not alone in this endeavour. In the dozens of manuals he published, the astrologer–physician Yang Shushan 袁樹珊 (1881–c. 1960s) did not only place Chinese astrology into a global context, linking it with European and American traditions, but also tried to explain its efficacy in relation to the latest studies in psychology, astronomy and spiritualism.^127^ Whereas in his influential *New Meanings on Geography*, Yu Renyu 俞仁宇 tried to clarify the arcane principles of traditional fengshui in light of such modern disciplines as electrical engineering, mineralogy and meteorology,^128^ Pan Ziduan 潘子端 (1902–1990) was among the earliest fortune-tellers who looked at Chinese divination through the lens of Carl G. Jung’s theory of the collective unconscious.^129^ By unearthing the alleged scientific principles hidden in traditional occult texts, Chinese occultists aimed to reinforce the usefulness of their arts in the modern world. Science not only became a powerful tool for legitimacy but also a borderline between ‘modern occultists’—who made secret knowledge public and open to scrutiny—and ‘traditional peddlers engaged in superstition’, who continued to envelope their arts in secrecy.^130^

## 6. Final Remarks

Past studies have amply recognised the early twentieth century as the period during which the persecution of occultists and folk healers reached unprecedented levels. Between 1927 and 1937, the Nanjing-based government issued numerous bans against ‘superstitious practices’, which included healing talismans, astrology, divination, hypnosis, and anything else related to the occult.^131^ Only a few historians, however, have acknowledged the social reactions these extreme measures sparked. Republican-era newspapers and printed books reveal that official bans on occult arts were not received without resistance. In 1928, a piece for *The Crystal* praises the China and West Press for standing up against the ‘tyrannical policies’ of the newly established Nanjing government. Titled ‘Why to admire the China and West Press?’, the article celebrates the remarkable courage of this tiny publisher, which decided to keep producing occult manuals despite political threats from the powerful central government. As the piece boldly declares, ‘the more you try to prohibit [occult arts], the more we will promote [them]’.^132^

To many Republican Chinese, their engagement with occult matters became an instrument of subversion against political tyranny, radical secularism and scientific materialism. In terms of healthcare, while they criticised Western biomedicine as dangerous, invasive and expensive, they also recognised that Chinese therapies like herbs and acupuncture could sometimes do more harm than good. Working on the same basis as the revolutionary hypnosis, talismans were yet cheap, effective and purely Chinese. Not only did they belong to China’s ‘national essence’, but their efficacy could be read in light of the latest Western and Japanese studies in psychology and faith healing. Simply put, talismans were both traditional and modern, local and universal, spiritual and scientific.

All this challenges simplistic Western views of modernity conceived of as secularism, materialism and a radical break with tradition. Have the Chinese ‘ever been modern’? The fact that the occult has never left Hong Kong and Taiwan’s public sphere—and that it has re-flourished in mainland China over the past two decades^133^ – demonstrates that disenchantment is not a universalising tendency of modernity but rather a continuum process that operates alongside that of (re)enchantment. Magic has not disappeared from modern life but is rather transformed and reinvented.^134^ The resilience of occult healing in modern Chinese society might disappoint some yet provide much food for thought to others.

Whereas new printing technologies and mass media had an enormous impact on the formation of a market of the occult in the Republican period, the popularisation of transnational spiritualism was decisive to augment and legitimise the interest Chinese elites had long attached to occult healing. Western and Japanese psychical researchers—many of whom were among the brightest scientific minds of their time—helped convince their contemporary Chinese fellows that the occult could also constitute an object of scientific inquiry. This gave rise to ‘Spiritual Science’ (*xinling kexue* 心靈科學) in the early twentieth century, a Sino-Japanese discipline inspired by the Anglo ‘psychical research’ and whose chief concerns lay in the scientific study of the mind, paranormal phenomena and psychic abilities—within which talismans and other Chinese occult arts were included. Indeed, the revival of the occult in the early twentieth century owed much of its success to the concurrent birth of the ‘mind’ in Chinese epistemology.^135^ In the end, although talismanic healing was not incorporated into TCM, claims that it should be appreciated as an indigenous technique of psychotherapy are still echoing today.^136^

To investigate the occult and make it useful to a ‘modern China’, secrets should first be systematised and disclosed to the public.^137^ Anyone should have the chance to try occult arts themselves without the restrictions imposed by esoteric lineages. The publication of self-learning manuals did not replace master-disciple relationships, divine inspiration or esoteric manuscripts, though. Although Yu Zhefu assured that the secrets revealed in his *Compendium* were enough to make talismans effective and that a ‘master was no longer necessary’ (*wushi zitong* 無師自通), for years he used the *Compendium* as a textbook in the society where he presided as director. But the publication of secrets did launch new ways for accessing hitherto occult knowledge. As Yang Der-Ruey has shown in his ethnographic study of a Daoist college in contemporary Shanghai, knowledge that had for centuries circulated within individual Daoist families are now being made public and shared by all students, both Daoist and laypeople, many of whom no longer recognise the secrecy within which that knowledge was once enveloped.^138^

Focused on case studies around talismanic healing, this article aimed to demonstrate the new lease of life the occult enjoyed during the first half of the twentieth century. It corroborates recent studies that show the alleged ‘marginalisation’ of occult healing in modern China more as a political ideal rather than an established fact.^139^ Further research on Republican occultism, the formation of Spiritual Science and the impact of occult arts in early twentieth-century China’s healthcare market are desperately needed. Another area that deserves attention is how Western and Japanese hypnosis provided a framework through which to clarify and validate the ‘scientific efficacy’ of Chinese occult healing arts. As Vivienne Lo has pointed out in 2009, new primary sources and methodologies have challenged conventional definitions of ‘Chinese medicine.’^140^ This article has indicated the enormous potential newspapers have for shedding light on hitherto neglected subjects in Chinese medical history, like healthcare technologies of everyday life and the activities of occultists who have left no written records other than a few advertisements.

## Footnotes

1 ‘Zhuyou zhibing (Talismanic Healing)’, *Xinwenbao* (Shanghai News), 4 June 1894, 3.

2 Zhu Min, ‘Zhibing? Pianren? Zhuyouke ji qita’, *Wanxiang zhoukan*, *111*, 4 August 1945, 2.

3
*Ibid*.

4 Luis Fernando B. Junqueira, ‘*Mingqing zhuyou zhi wenti kaoshi* (The Debates around the Origin of Talismanic Healing in the Ming and Qing Dynasties)’ (Master’s thesis, Fudan University, 2018).

5 Joseph Needham, *Clerks and Craftsmen in China and the West* (Cambridge: Cambridge University Press, 1970), 268.

6 Li Jingwei and Zhang Zhibin, eds, *Zhongyixue sixiangshi* (Changsha: Hunan jiaoyu chubanshe, 2006), 296–319.

7 Volker Scheid, *Currents of Tradition in Chinese Medicine, 1626-2006* (Seattle: Eastland Press, 2007); Kim Taylor, *Chinese Medicine in Early Communist China, 1945–1963: A Medicine of Revolution* (London: Routledge, 2005). Although Lei and Andrews have slightly touched upon the place of occult arts in twentieth-century China, the creation of Traditional Chinese Medicine still dominates their discussion. Bridie Andrews, *The Making of Modern Chinese Medicine, 1850-1960* (Vancouver: UBC Press, 2015); Sean Hsiang-Lin Lei, *Neither Donkey nor Horse: Medicine in the Struggle over China’s Modernity* (Chicago: University of Chicago Press, 2014).

8 Other common terms are *fangshu* 方術 (methods and calculations) and *yibu xingxiang* 醫卜星相 (medicine, divination, astrology and physiognomy). Richard J. Smith, ‘Divination: Science, Technology, and the Mantic Arts in Traditional China’, in Helaine Selin, ed, *Encyclopaedia of the History of Science, Technology, and Medicine in Non-Western Cultures* (Dordrecht: Springer, 2008), 684–692.

9 Donald Harper and Marc Kalinowski, ‘Introduction,’ in Donald Harper and Marc Kalinowski, eds, *Books of Fate and Popular Culture in Early China: The Daybook Manuscripts of the Warring States, Qin, and Han* (Leiden: Brill, 2017), 5.

10 Kristofer M. Schipper, *The Taoist Body* (Berkeley: University of California Press, 1993), xii-xiii, 2–18.

11 Yoshinaga Shin’ichi, ‘Seishin no chikara: Minkan seishinryōhō no shisō’ (The Birth of Japanese Mind-Cure Methods), *Ningen* kagaku, 2007, 16, 9–21.

12 Wouter J. Hanegraaff, ‘How Magic Survived the Disenchantment of the World’, *Religion*, 2003, *33*, 357–380.

13 Rebecca Nedostup, *Superstitious Regimes: Religion and the Politics of Chinese Modernity* (Cambridge, MA: Harvard University Asia Center, 2009).

14 Wen-Hsin Yeh, *Shanghai Splendor: Economic Sentiments and the Making of Modern China, 1843-1949* (Berkeley: University of California Press, 2007).

15 Jing Tsu and Benjamin A. Elman, eds, *Science and Technology in Modern China, 1880s–1940s* (Leiden: Brill, 2014); Philip Clart, David Ownby and Chien-chuan Wang, eds, *Text and Context in the Modern History of Chinese Religions: Redemptive Societies and Their Sacred Texts* (Leiden: Brill, 2020).

16 Chen Duxiu, ‘Jinri Zhongguo zhi zhengzhi wenti (Political Issues in Today’s China)’, *Xin Qingnian* (New Youth), 15 July 1918.

17 Yu Pingke, ‘Yanjiu xinlingxue shi yizhong mixin de gongzuo me (Is Spiritual Science a Superstitious Endeavour?)’, *Xinling wenhua* (Spiritual Culture), 1931, 9–10.

18 Christopher A. Reed, *Gutenberg in Shanghai: Chinese Print Capitalism, 1876–1937* (Vancouver: UBC Press, 2004).

19 By ‘spiritualism’ I also refer to psychology and psychical research more broadly. On the intertwined connection between these three, see Andreas Sommer, ‘Crossing the Boundaries of Mind and Body: Psychical Research and the Origins of Modern Psychology’ (PhD diss., University College London, 2013), 60–127, 139–146; Richard Noakes, *Physics and Psychics: The Occult and the Sciences in Modern Britain* (Cambridge: Cambridge University Press, 2019); Yoshinaga Shin’ichi, *Nihonjin no mi-shin-rei: Kindai minkan seishinryōhō sōsho* (Body-Mind-Spirit: Folk Mind-Cure Methods in Modern Japan), 2 vols (Tokyo: Kuresushuppan, 2004), II, 1–17.

20 Philip Clart, ‘New Technologies and the Production of Religious Texts in China, 19th to 21st Century’, in Vincent Goossaert, Jan Kieley and John Lagerwey, eds, *Modern Chinese Religion II, 1850-2015*, 2 vols (Leiden: Brill, 2016), I, 560–78; Gregory A. Scott, ‘Conversion by the Book: Buddhist Print Culture in Early Republican China’ (PhD diss., Columbia University, 2013).

21 Lauren Kassell, ‘The Economy of Magic in Early Modern England’, in Margaret Pelling and Scott Mandelbrote, eds, *The Practice of Reform in Health, Medicine, and Science, 1500–2000: Essays for Charles Webster* (Aldershot: Ashgate, 2005), 43–57.

22 Yu Zhefu, *Shenmi fuzhou quanshu*, 4 vols (Shanghai: Chenzhou jingling xueshe, 1922).

23 Yu Zhefu, *Fuzhou yanjiufa* (Shanghai: Chenzhou jingling xueshe, 1924).

24
*Ibid*., preface.

25 Junqueira, ‘*Mingqing zhuyou*’, 41–45.

26 Here, the characters of the term *zhuyou* read ‘[the art] the Zhu 祝 [clan] originated (*you* 由)’.

27 Ding Rouke. *Liuhu* (The Willow Bow), 6 vols (Beijing: Zhonghua shuju, 2002), II. The Zhu clan is probably a reference to Zhurong 祝融, an ancient god of fire related to the healing of wounds and fractures by talismans. See Junqueira, ‘*Mingqing zhuyou*’, 21–22, 41, 65.

28 Donald Harper, *Early Chinese Medical Literature: The Mawangdui Medical Transcripts* (London: Routledge, 1998), 159–183.

29
*Xinwenbao*, 28 April 1922.

30 Angela Ki Che Leung, ‘Charity, Medicine, and Religion: The Quest for Modernity in Canton (ca. 1870–1937)’, in *Chinese Modern Religion II: 1850–2015*, II, 579–812; Fan Li and Michael Lackner, ‘Contradictory Forms of Knowledge? Divination and Western Knowledge in Late Qing and Early Republican China,’ in Michael Lackner, ed, *Coping with the Future: Theories and Practices of Divination in East Asia* (Leiden: Brill, 2017), 451–485.

31 On spiritualism and psychical research in fin-de-siecle Japan, see Ichiyanagi Yoshitaka, *Kokkurisan to senrigan: Nihon kindai to shinreigaku* (Table-Turning and Telepathy: Spiritualism in Modern Japan) (Tokyo: Kodansha, 1994); Yoshinaga Shin’ichi, ed, *Saiminjutsu no reimei: Kindai nihon rinshō shinri no tanjō* (The Dawn of Hypnosis: The Birth of Experimental Psychology in Modern Japan) (Tokyo: Kuresu shuppan, 2006).

32 See, for example, Douglas M. Gildow, ‘Cai Yuanpei (1868–1940), Religion, and His Plan to Save China through Buddhism,’ *Asia Major*, 2018, *31.2*, 107–148; Li and Lackner, ‘Contradictory Forms of Knowledge?’.

33
*Xinwenbao*, 28 April 1922.

34
*Xinwenbao*, 17 May 1922.

35
*Xinwenbao*, 11 June 1922.

36 The claim for ‘revealing secrets’ also grew in early-modern England. Elaine Leong and Alisha Rankin, eds, *Secrets and Knowledge in Medicine and Science, 1500–1800* (New York: Routledge, 2011).

37
*Xinwenbao*, 17, 21, 24, 26, 29 May 1924.

38 This is based on a collection of around 300 talismanic texts I collected or consulted in China between 2014 and 2019. This number can be corroborated by the talismanic texts kept at the Berlin State Library, most of which are manuscripts. Paul U. Unschuld and Zheng Jinsheng, *Chinese Traditional Healing: The Berlin Collections of Manuscript Volumes from the 16th through the Early 20th Century* (Leiden: Brill, 2012).

39 See, for example, Zheng Yuxiang, *Fulu zhuyou* (Healing Talismans) (Taichung: Ruicheng shuju, 2017).

40 Yu, *Fuzhou yanjiufa*, 1.

41
*Ibid*., 5.

42
*Shenbao* (Shanghai Daily), 13 July 1924.

43
*Ibid*.

44
*Shenbao*, 4 June 1923.

45
*Shenbao*, 25 September 1922.

46
*Shenbao*, 28 May 1924.

47 For the term *zhengming*, see Erik J. Hammerstrom, *The Science of Chinese Buddhism: Early Twentieth-Century Engagements* (New York: Columbia University Press, 2015), 82–84.

48 The title of the section on healing talismans, for example, is written *Zhuyou yanjiu* (Research on Talismanic Healing). The same applies to the other 10 entries.

49 Yu, *Fuzhou yanjiufa*, 1.

50 Lu Kefeng, ‘Zhongguo cuimianshu’, *Dongfang zazhi* (Eastern Miscellany), 1917, *14.2–3*, 91–96, 85–91.

51
*Xinwenbao*, 9, 15 August 1924.

52 Hanegraaff, ‘How Magic Survived.’

53 Sommer, ‘Crossing the Boundaries’; Noakes, *Physics and Psychics*; Yoshinaga, *Nihonjin no mi-shin-rei*.

54 Yu, *Cuimianshu*, 41–50.

55 Yoshinaga, ‘Seishin no chikara’; Yu-chuan Wu, ‘The Moral Power of Suggestion: A History of Suggestion in Japan, 1900-1930’, *Journal of the History of the Behavioral Sciences*, 2019, *55*, 21–39.

56 Alex Owen, *The Place of Enchantment: British Occultism and the Culture of the Modern* (Chicago: University of Chicago Press, 2004), 1–30; Sommer, ‘Crossing the Boundaries’, 137–98.

57 Matthias Schumann, ‘Science and Spirit-Writing: The Shanghai Lingxuehui and the Changing Fate of Spiritualism in Republican China,’ in *Text and Context*, 126–72.

58 Yu Pingke, *Cuimianshu hanshou jiangyi* (Lectures on Hypnosis for Distance Learning) (Shanghai: Xinling kexue shuju, 1931).

59
*Xinwenbao*, 26 June 1924.

60
*Xinwenbao*, 21 August 1924.

61 On ‘experience’ as a new epistemological category in modern China, see Sean Hsiang-lin Lei, ‘How Did Chinese Medicine Become Experiential? The Political Epistemology of Jingyan’, *Positions: East Asian Cultures Critique*, 2002, *10*, 333–64; Emily Baum, *The Invention of Madness: State, Society, and the Insane in Modern China* (Chicago: University of Chicago Press, 2018), 97–103.

62 See, for example, Xie Lujun, ed, *Shuzang* (Collection of Secret Arts), 100 vols (Beijing: Yanshan chubanshe, 2010); Guojia tushuguan diancang yuedubu, ed, *Minguo shiqi faxing shumu huipian* (Collection of Publishers’ Catalogues from the Republican Period), 20 vols (Beijing: Guojia tushuguan chubanshe, 2010).

63 Reed, *Gutenberg in Shanghai*, 173–245.

64 Zhongxi shuju, *Shanghai Zhongxi shuju tushu mulu* (Title Catalogue of the China and West Press) (Shanghai: Zhongxi shuju, 1927).

65
*Xinwenbao*, 29 May 1926.

66
*Xinwenbao*, 29 November 1929.

67 Zhongxi shuju, *Shanghai Zhongxi shuju*, 11–13.

68
*Xinwenbao*, 30 September 1928.

69 Guoyi xueshe and Xingming yanjiushe, eds, *Yibu xingxiang bairitong* (Learn Medicine, Divination, Astrology and Physiognomy in 100 Days) (Shanghai: Zhongyang shudian, 1937); Guoyi xueshe, ed, *Jianghu yishu mizhuan* (Secret Transmissions from Folk Healers) (Taichung: Ruicheng shuju, 1980).

70
*Xuanyuan beiji zhuyou shisanke* (Shanghai: Guangyi shuju, 1914).

71
*Ibid*.,16.

72 Xu Jinghui, *Zhuyouke zhibing qishu* (Shanghai: Zhongxi shuju, 1927), 12.

73 Unschuld and Zheng, *Chinese Traditional Healing*, 18–56, 154–76.

74 Ichiyanagi Yoshitaka, *Saiminjutsu no nihon kindai* (Hypnosis in Modern Japan) (Tokyo: Seikyūsha, 1997); Alan Gauld, *A History of Hypnotism* (Cambridge: Cambridge University Press, 1992).

75 Zheng Guo, *Biandong shehuixia de xinyang fenhua Shanghai Lingxuehui yanjiu* (The Shanghai Spiritualist Society) (Beijing: Zhongguo shehui kexue chubanshe, 2018), 18–51.

76 Ouyang Zhongshou, ‘Second preface’, in Guo Renlin, *Yougui lun* (Shanghai: n.p, 1917).

77 That is the Wangping Street (望平街), popularly known as the Newspaper Street (報館街) during the Republican period.

78 Zhongxi shuju, *Shanghai Zhongxi shuju*, n.p.

79 The database *Quanguo baokan suoyin* (National Index to Chinese Newspapers and Periodicals) records over 150 articles titled ‘*yuanguang*’ published between 1910 and 1939.

80 Elmer E. Knowles, *The Basic Principles of Suggestion, Hypnotism, Telepathy, Personal Magnetism, Character Building … and the Development of the Dormant Faculties* (Brussels: Psychology Foundation, 1926).

81 Zhongxi shuju, *Shanghai Zhongxi shuju*, n.p.

82
*Ibid*.; *Xinwenbao*, 1 May 1929.

83 Yu, *Cuimianshu*, 41–50; Lu, ‘*Zhongguo cuimianshu*’.

84 Gauld, *A History of Hypnotism*, 273–503.

85 Zheng, *Biandong shehuixia*, 18–51.

86 Buyi, ‘Zhongguo zhuyouke zai jingliao xueshu shang zhi pingjia’ (Comments on the Place of Chinese Talismans in the Academic Field of Psychotherapy), *Jiankang zazhi* (Health Magazine), 1937, 5, 30–31.

87 Li Xianghou, ‘*Jingshen youxi zhuyouke* (Psychological Games: Talismanic Healing)’, *Jingshen yuekan* (Monthly Psychic), 1918, *2*, 23–24; Feng, ‘Shenmi de zhuyouke (Mysterious Talismanic Healing)’, *Sanliujiu huabao* (369 Illustrated Magazine), 1942, 14, 19.

88 Buyi, ‘Zhongguo zhuyouke’.

89 Wang Yang, *Zhongxi cuimianshu jiangyi* (Shanghai: Zhongxi yiyuan, 1924).

90 Huang Feng, *Shijie cuimianshu daquan* (Shanghai: Guoguang shudian, 1940).

91
*Xinwenbao*, 30 September 1929.

92 For their Chinese names, please refer to Table 1.

93 Zheng, *Biandong shehuixia*; Wang Chien-ch’uan, ‘Spirit Writing Groups in Modern China (1840–1937): Textual Production, Public Teachings, and Charity’, in *Chinese Modern Religion II: 1850–2015*, II, 651–84.

94 Owen Davies, ‘Newspapers and the Popular Belief in Witchcraft and Magic in the Modern Period’, *Journal of British Studies*, 1998, 37, 139–65.

95
*Xinwenbao*, 30 April 1922.

96
*Shibao* (Eastern Times), 10 June 1920.

97
*Xinwenbao*, 25 May 1925.

98
*Shenbao*, 29 July 1923.

100 Luis Fernando B. Junqueira, ‘Popular Healing in Printed Medical Books: The Compilation and Publication of the Chuanya from the Late Qing through the Republican Period’, *Monumenta Serica*, 2018, 66, 391–436; Paul U. Unschuld, ‘Chinese Retributive Recipes: On the Ethics of Public and Secret Health Care Knowledge’, *Monumenta Serica*, 2004, 52, 325–43.

101
*Xinwenbao*, 8 October 1922.

102
*Xinwenbao*, 6 February 1895.

103
*Xinwenbao*, 7 August 1918.

104
*Xinwenbao*, 28 May 1915.

105
*Shenbao*, 17 May 1924.

106
*Xinwenbao*, 27 May 1925.

107
*Xinwenbao*, 26 November 1921.

108
*Xinwenbao*, 10 October 1932.

109
*Shenbao*, 31 October 1914.

110
*Shenbao*, 30 August 1916.

111
*Shenbao*, 10 April 1926.

112
*Xinwenbao*, 22 October 1925.

113
*Fu’er mosi* (Holmes), 16, 20, 26 August 1927.

114
*Fu’er mosi*, 30 November 1932.

115
*Shenbao*, 21 December 1926.

116 For the statistics, see entries for *zhuyou*, *fuzhou* and *lingfu* in the database *Quanguo baokan suoyin*.

117 Corinna Treitel, *A Science for the Soul: Occultism and the Genesis of the German Modern* (Baltimore: John Hopkins University Press, 2004), 56–82.

118
*Shenbao*, 16 January 1925.

119
*Shenbao*, 23 March 1923.

120
*Shenbao*, 28 May 1923.

121
*Shenbao*, 4 October 1926.

122 David A. Palmer, ‘Chinese Redemptive Societies and Salvationist Religion: Historical Phenomenon or Sociological Category?’, *Journal of Chinese Ritual, Theatre and Folklore*, 2011, 172, 21–72.

123 Tan Yangwu, *Tanshi sanyuan dili daxuankong lutou* (Shanghai: Sanyuan qishu yanjiushe, 1923), personal preface.

124
*Shenbao*, 2 December 1922.

125
*Shenbao*, 16 February 1925.

126
*Shenbao*, 7 March 1941.

127 Yuan Shushan, *Shu bushixingxiangxue* (Studies on Divination and Astrology) (Zhenjiang: Rundetang, 1934).

128 Yu Renyu, *Dilixue xinyi* (Shanghai: Wenming shuju, 1935).

129 Pan Ziduan, *Mingxue xinyi* (Shanghai: Wenming shuju, 1939).

130 The same demarcation took place in the field of Chinese divination. Fan and Lackner, ‘Contradictory Forms of Knowledge?’.

131 Nedostup, *Superstitious Regimes*, 150–226.

132
*Dajingbao*, 13 July 1928, 3.

133 Adam Y. Chau, *Miraculous Response: Doing Popular Religion in Contemporary China* (Stanford: Stanford University Press, 2008); Ole Bruun, *Fengshui in China: Geomantic Divination Between State Orthodoxy and Popular Religion* (Honolulu: University of Hawaii Press, 2003).

134 Hanegraaff, ‘How Magic Survived’; Richard Jenkins, ‘Disenchantment, Enchantment and Re-Enchantment: Max Weber at the Millennium’, *Max Weber Studies*, 2000, 1, 11–32.

135 I am grateful to one of the anonymous reviewers for pointing this out. See also Baum, *The Invention of Madness*, especially chapter 4.

136 See, for example, Wang Zhengkai and Liu Mingjun, ‘Zhuyou yuanshi de xinli zhiliao fangfa’ (Talismanic Healing and the Origins of Psychotherapy), *Zhonghua zhongyiyao zazhi*, 2017, *8*, 3653–56.

137 This is also evident in the case of the physician-astrologer Yuan Shushan. Li and Lackner, ‘Contradictory Forms of Knowledge?’.

138 Yang Der-Ruey, ‘Revolution of Temporality: The Modern Schooling for Daoist Priests in Contemporary Shanghai,’ in David Palmer and Xun Liu, eds, *Daoism in the Twentieth Century* (Berkeley: University of California Press, 2012), 67.

139 David Palmer, *Qigong Fever: Body, Science, and Utopia in China* (New York, Columbia University Press, 2007); Xun Zhou, *The People’s Health: Health Intervention and Delivery in Mao’s China 1949-1983* (Montreal: McGill-Queen’s University Press, 2020).

140 Vivienne Lo, ‘But is it [History of] Medicine? Twenty Years in the History of the Healing Arts of China,’ *Social History of Medicine*, 2009, 22.2, 283–303.

99
*Shenbao*, 22 July 1929.

## Notes

Luis Fernando Bernardi Junqueira (林友樂) has earned a Master’s Degree in Chinese History at Fudan University and is currently a PhD student in the Department of History at University College London. Funded by the Wellcome Trust, his PhD research investigates the transnational history of psychology, psychical research and the occult in early twentieth-century China through the emergence of ‘Spiritual Science’ (*xinling kexue*), looking at its impact on healthcare and religious experience. His research interests include Chinese medical history, science and technology studies, occultism, print culture and transnational history. He has published in English, Chinese and Portuguese.

